# Impact of absolute values and changes in meteorological and air quality conditions on community-acquired pneumonia in Germany

**DOI:** 10.1007/s00484-024-02839-7

**Published:** 2024-12-24

**Authors:** Saeed A. Khan, Thomas Brenner, Ann-Christine Link, Christoph Reudenbach, Jörg Bendix, Barbara C. Weckler, Max Kutzinski, Jan Rupp, Martin Witzenrath, Gernot Rohde, Mathias W. Pletz, Wilhelm Bertrams, Bernd Schmeck

**Affiliations:** 1 Department of Geography, Philipps-Universität Marburg, Marburg, Germany; 2 Institute for Lung Research, German Center for Lung Research (DZL), Universities of Giessen and Marburg Lung Centre, Philipps-Universität Marburg, Marburg, Germany; 3 Department of Medicine, Pulmonary and Critical Care Medicine, University Medical Center Marburg, Philipps-Universität Marburg, German Center for Lung Research (DZL), Marburg, Germany; 4 CAPNETZ STIFTUNG, Hannover, Germany; 5 Department of Infectious Diseases and Microbiology, University Hospital Schleswig-Holstein, Lübeck, Germany; 6 Department of Infectious Diseases, Respiratory Medicine and Critical Care, Charité, Universitätsmedizin Berlin, Berlin, Germany; German Center for Lung Research (DZL), Berlin, Germany; 7 Department of Respiratory Medicine, Goethe University Frankfurt, University Hospital, Medical Clinic I, Frankfurt, Germany; 8 Biomedical Research in End-stage and Obstructive Lung Disease Hannover (BREATH), Member of the German Center for Lung Research (DZL), Hannover, Germany; 9 Institute for Infectious Diseases and Infection Control, Jena University Hospital and Friedrich-Schiller-University Jena, Jena, Germany; 10 German Center for Infection Research (DZIF), the Center for Synthetic Microbiology (SYNMIKRO), Marburg, Germany, and the Institute for Lung Health (ILH), Giessen, Germany

**Keywords:** Climate change, Pneumonia, Weather, Health, Air pollution, Hospitalization

## Abstract

Community-acquired pneumonia (CAP) is a major global health concern as it is a leading cause of morbidity, mortality and economic burden to the health care systems. In Germany, more than 15,000 people die every year from CAP. Climate change is altering weather patterns, and it may influence the probability and severity of CAP. The increasing frequency and intensity of extreme weather events necessitate the study of their impact on CAP hospitalizations. In this regard, we examine the influence of absolute values and changes in various meteorological and air quality conditions on the frequency of CAP hospitalizations. We matched weather data to the German CAPNETZ-Cohort of 10,660 CAP patients from 22 healthcare facilities between 2003 and 2017. Our findings show that daily fluctuations in meteorological conditions (maximum temperature, 99th percentile precipitation), apart from relative humidity, are likely to result in triggering CAP hospitalizations than absolute conditions. In contrast, the absolute values of air quality (CO, NO_2_, O_3_, PM_2.5_, SO_2_, and total aerosols) are found to exert a greater influence on CAP hospitalizations than changes in air quality. The study outcomes have implications for public health measures, early warning systems, and public awareness, with the aim of mitigating the risk of CAP.

**Supplementary Information:**

The online version contains supplementary material available at 10.1007/s00484-024-02839-7.

## Introduction

Community-acquired pneumonia (CAP), an acute infection of the pulmonary parenchyma that occurs in individuals outside of healthcare settings, is a significant global health concern (Aliberti et al. 2021). Specific demographic groups, including the elderly, children, and those with comorbidities, are particularly vulnerable to developing CAP infections (Brenner et al. 2024; Cardinale et al. 2013; Cillóniz et al. 2018, 2020).

Climate change presents a substantial threat to global health in two principal ways: through slow-onset changes in weather patterns and through the increased occurrence of rapid-onset extreme weather events, both of which are projected to increase in severity and frequency (Butsch et al. 2023). In addition, exposure to particulate matter and other air pollutants is associated with increased morbidity and mortality in connection with CAP (Lee et al. 2021; Lin et al. 2023).

Existing literature focuses primarily on the relationship between temperature and CAP admission (Guo et al. 2021; Kapwata et al. 2021; Tian et al. 2023), while other meteorological and air quality conditions have received little attention, except for Wang et al. (2022), which examined associations between air pollutants and pneumonia admissions in China. Research investigating the influence of sudden changes in weather conditions from one day to the next is limited. Notable exceptions include investigations of the effects of temperature changes from the previous day to the current day on pneumonia-related hospitalizations in South Korea (Sohn et al. 2019), of temperature changes and mortality causes (cardiovascular and respiratory) in the United States (Zhan et al. 2017), and of ambient temperature variability and pneumonia admissions in China (Tian et al. 2023). However, a comparison between the impact of absolute values (conditions on a specific day before hospitalization) and changes in weather conditions (value of the specific day before hospitalization minus the previous day’s) on CAP risk is currently missing. In this paper, we conduct such a comparison for CAP hospitalizations, including both in-patients and out-patients’ clinics in the German multi-center CAPNETZ-Cohort, a dataset of individuals hospitalized for pneumonia. By this, we examine for each meteorological and air quality condition separately whether the absolute values or day-to-day changes matter.

## Materials and methods

**Fig. 1:**
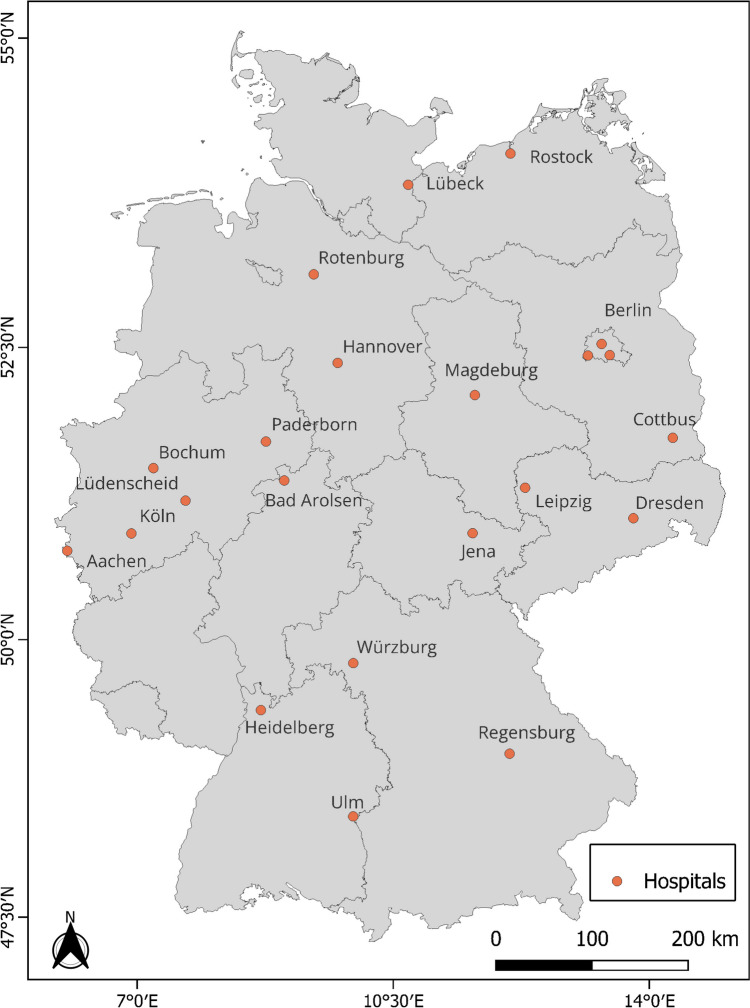
Map showing the locations of 22 clinics and hospitals in the CAPNETZ clinical dataset used for this study

This study uses the CAPNETZ clinical dataset encompassing 10,660 individuals hospitalized for pneumonia in 22 hospitals and medical practices between 2003 (the start of weather data availability) and 2017 (the end of CAPNETZ 1.0 data acquisition) (NCT 02139,163). The geographical locations of the clinics and hospitals are shown in Fig. 1, while information on the number of cases, the period considered, coordinates, and altitude is provided in Table 1 (Supplementary Material).

A detailed description of the CAPNETZ methodology is provided by Welte et al. (2004). Patients eligible for inclusion were those aged 18 or older with pulmonary infiltrates confirmed by chest imaging and at least one of the following: fever (≥ 38.3 °C), cough, purulent sputum production, or focal chest signs on auscultation. Exclusion criteria included acquired or therapeutically induced immune deficiency, active tuberculosis, or nosocomial infection acquisition. Clinical data was electronically recorded, noting the exact hospitalization date. Patients were followed up for 180 days using a standardized protocol. Outcomes were assessed through structured in-person or phone interviews with the patients or their relatives. Written informed consent was obtained from each patient, and the study was approved by the local ethical committees of the centers participating in CAPNETZ, ensuring compliance with relevant guidelines and regulations.

A total of eleven meteorological and air quality conditions were analyzed: the meteorological conditions of air pressure, relative humidity, maximum temperature, and precipitation above the 99th percentile, and the air quality conditions of CO, NO_2_, O_3_, SO_2_, PM_2.5_, dust aerosol, and total aerosol. A brief description of these variables is provided in Table 2 (Supplementary Material). Most meteorological data was sourced from the German Weather Service (DWD). Quality controls were applied to ensure data consistency and accuracy. Terrain data from the Shuttle Radar Topography Mission (SRTM) was used to account for climatological effects and resampled to 500 × 500 m cells. Ordinary kriging with external drift was employed to interpolate daily climate data for each German municipality, using R-software and integrating SRTM terrain heights. Precipitation data were derived from the COSMO-REA6 reanalysis (6 × 6 km resolution) and extreme precipitation data from E-OBS (0.02° x 0.02° resolution). Data on air quality conditions came from the Copernicus Atmosphere Monitoring Service (0.75° x 0.75° resolution). The weather data was matched to each case based on hospital location and date. Weather conditions were assessed in municipalities within 1 h’s driving distance from the clinic, and then a weighted average was built based on population and distance using a distance decay function (Brenner and Pudelko 2019).

A logistic regression approach was used to examine which weather conditions influenced CAP hospitalizations. To this end, we generated five hypothetical random hospitalization dates (non-real cases) for each patient in our medical patient cohort dataset, assigning the same location but a different admission date to them.

The aim was to examine whether the weather conditions or their change better explain the dates of the real hospitalization cases. Therefore, we conducted separate logistic regressions for each weather condition and their day-to-day change as independent variables. Monthly dummies were added to the model to capture the seasonality of CAP. We calculate each regression’s Akaike information criterion (AIC) as a goodness of fit measure, allowing us to compare the relevance of each condition and its change. The AIC is also used to determine whether the conditions should be used for the day of hospitalization, or one, two, three, four, five, or six days before. The goodness of fit is best for the conditions from the day before hospitalization and the changes from two days before to the day before hospitalization. We did not test more than six days before hospitalization because the AIC signals a clearly less good fit for six days before than for one day before. As a robustness check, we repeated the analyses for conditions extending more than one day before hospitalization up to six days before hospitalization. The results do not change, except for two conditions. In the case of maximum temperature, we obtain different results for more than three days before hospitalization but with a clearly lower goodness of fit. In the case of O_3_, using different numbers of days before hospitalization leads to varying results, decreasing the reliability of the results for O_3_. Additional robustness checks include removing monthly dummies and separately analyzing rural and urban areas. We find three deviations from the results below: the comparison turns around for temperature and humidity if monthly dummies are removed, reflecting seasonality, and for dust aerosols in urban areas.

In all regressions, both linear and quadratic terms were analyzed, and the model leading to the lowest AIC was chosen. The log-likelihood ratio was calculated for each weather condition compared to a model without weather conditions. The log-likelihood ratio shows how much better a model with a weather condition can predict hospitalizations than a model without the weather condition. According to the likelihood-ratio test statistics (Woolf 1957; Lewis et al. 2011), a likelihood-ratio below 1 implies that the model without the weather conditions leads to the better fit (lower AIC) and the weather condition has no predictive power. Log-likelihood ratio values above 1 signal a better fit, and values above 1.92 indicate a significant contribution of weather conditions in the prediction of CAP hospitalizations. We use the log-likelihood ratio values to compare the contribution of absolute values and changes in weather conditions. While the likelihood ratio values are our main results, in some cases it is interesting to examine the logistic regression line, especially to detect differences between summer and winter conditions.

## Results and discussion

**Fig. 2:**
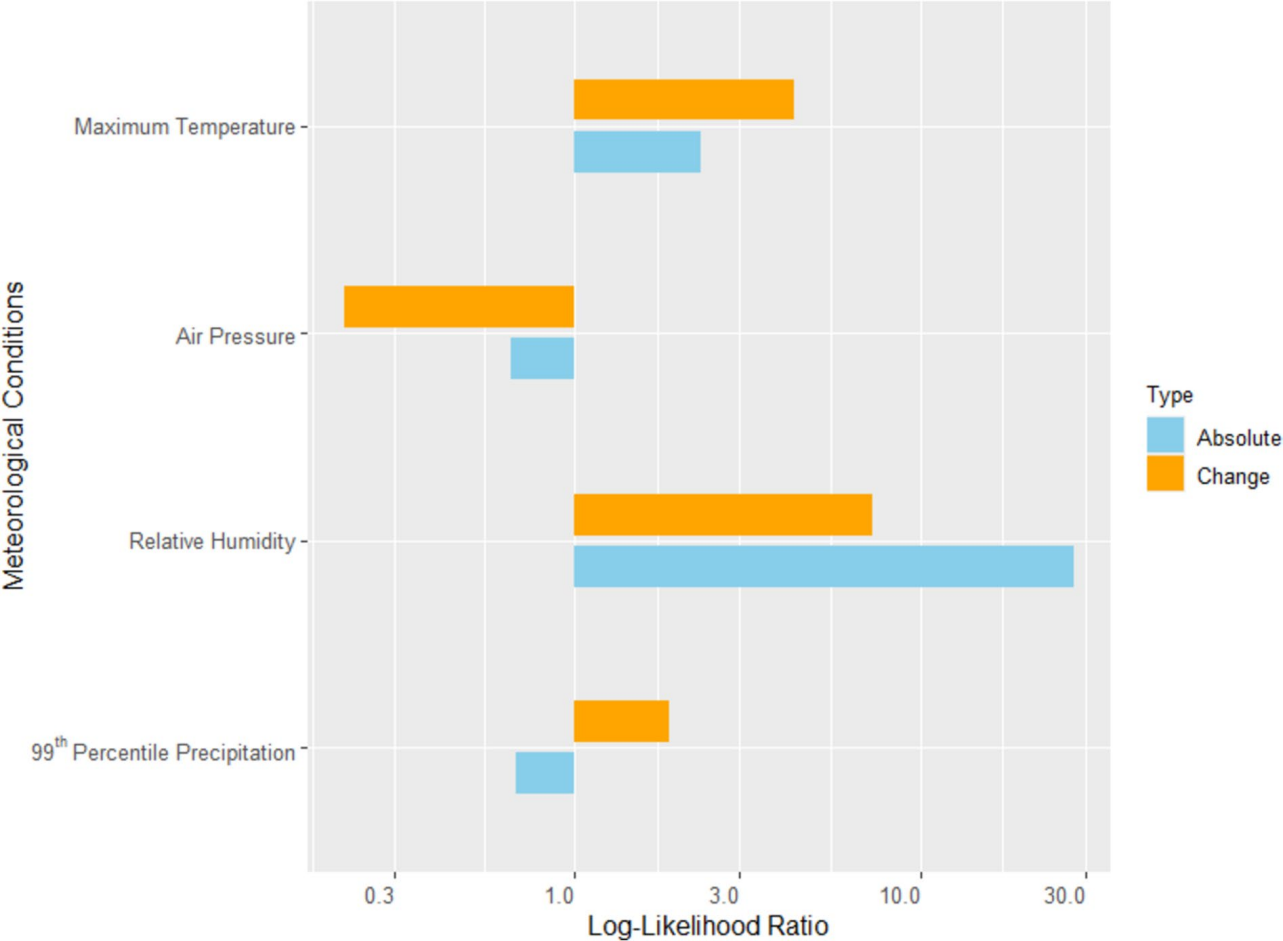
Comparison of the contribution to the CAP prediction by models with absolute conditions and changes in meteorological conditions using the log-likelihood ratio displayed in logarithmic scale

Our aim is to compare how much the absolute values and changes of meteorological and air quality conditions are related to CAP hospitalizations in Germany. Considering the meteorological conditions (Fig. 2), we find a stronger relationship with changes (maximum temperature and precipitation), as well as a stronger relationship with absolute values (relative humidity), while for air pressure, no significant relationship is found in any case.

**Fig. 3:**
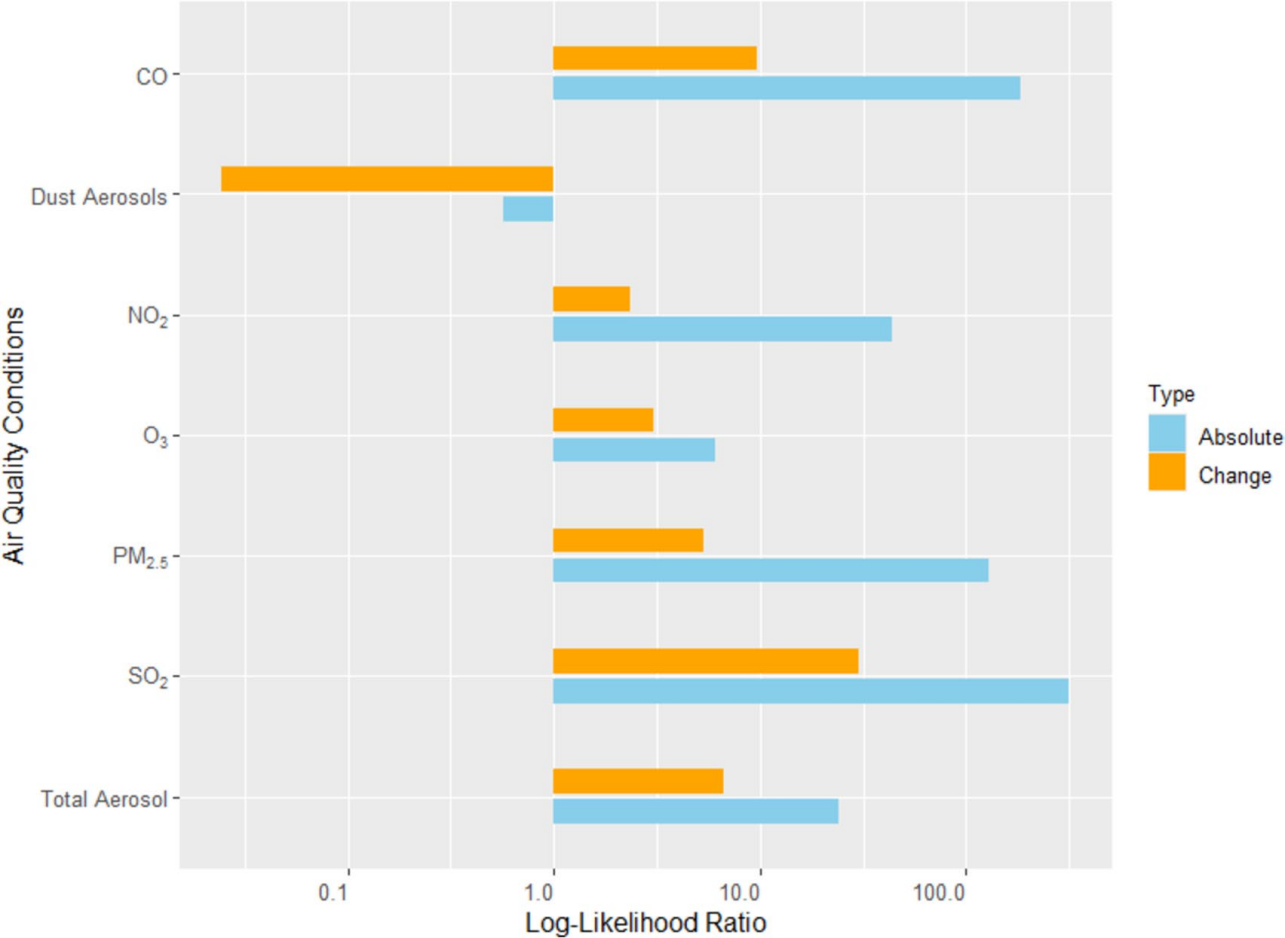
Comparison of the contribution to the CAP prediction by models with absolute conditions and changes in air quality conditions using the log-likelihood ratio displayed in logarithmic scale

The picture is more homogeneous for the air quality conditions (Fig. 3): the absolute levels are more strongly related to CAP hospitalization than the changes in air quality conditions. In the case of dust aerosols, a significant association between its absolute value and CAP hospitalizations is not found in the main analysis, but it is found for rural areas, which are studied as a robustness check.

Furthermore, the highest log-likelihood ratios are found for air quality conditions. This indicates that air quality conditions contribute more to CAP hospitalizations than meteorological conditions.

In the case of maximum temperature and extreme precipitation, our results indicate that sudden changes are more relevant for CAP hospitalization than continuously high values.

**Fig. 4:**
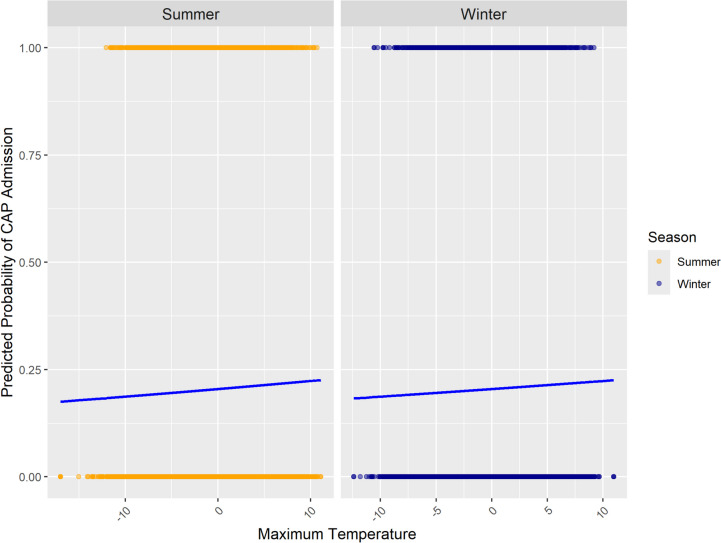
Predicted probabilities of CAP hospitalizations associated with changes in maximum temperature during winter and summer

These findings align with previous research highlighting the significance of meteorological variability in respiratory illnesses (Zhan et al. 2017; Sohn et al. 2019). Zhan et al. (2017) state that the human body may not readily adapt to sudden changes in weather conditions, such as humidity and precipitation as well as stronger changes in temperature, particularly in the case of chronic health problems. Furthermore, Tian et al. (2023) found that acute exposure to temperature variability leads to increased hospital admissions for pneumonia in China. Interestingly, our results confirm these findings for temperature and precipitation. In the case of relative humidity, we find a strong relationship between changes and CAP hospitalization, but an even stronger relationship between absolute values and CAP hospitalization. A potential explanation is that lasting high or low humidity levels support the survival of respiratory viruses (Davidse and Zare 2021) or that (lasting) low humidity is associated with increased respiratory tract infections (Mäkinen et al. 2009). Considering the logistic regression lines for temperature (Fig. 4) provides an interesting additional insight. It shows that the values of maximum temperature changes are quite similar in summer and winter. Our results have shown that the changes in temperature are more important than their absolute values, so that the effect of temperature on CAP hospitalization is quite the same in summer and winter.

**Fig. 5:**
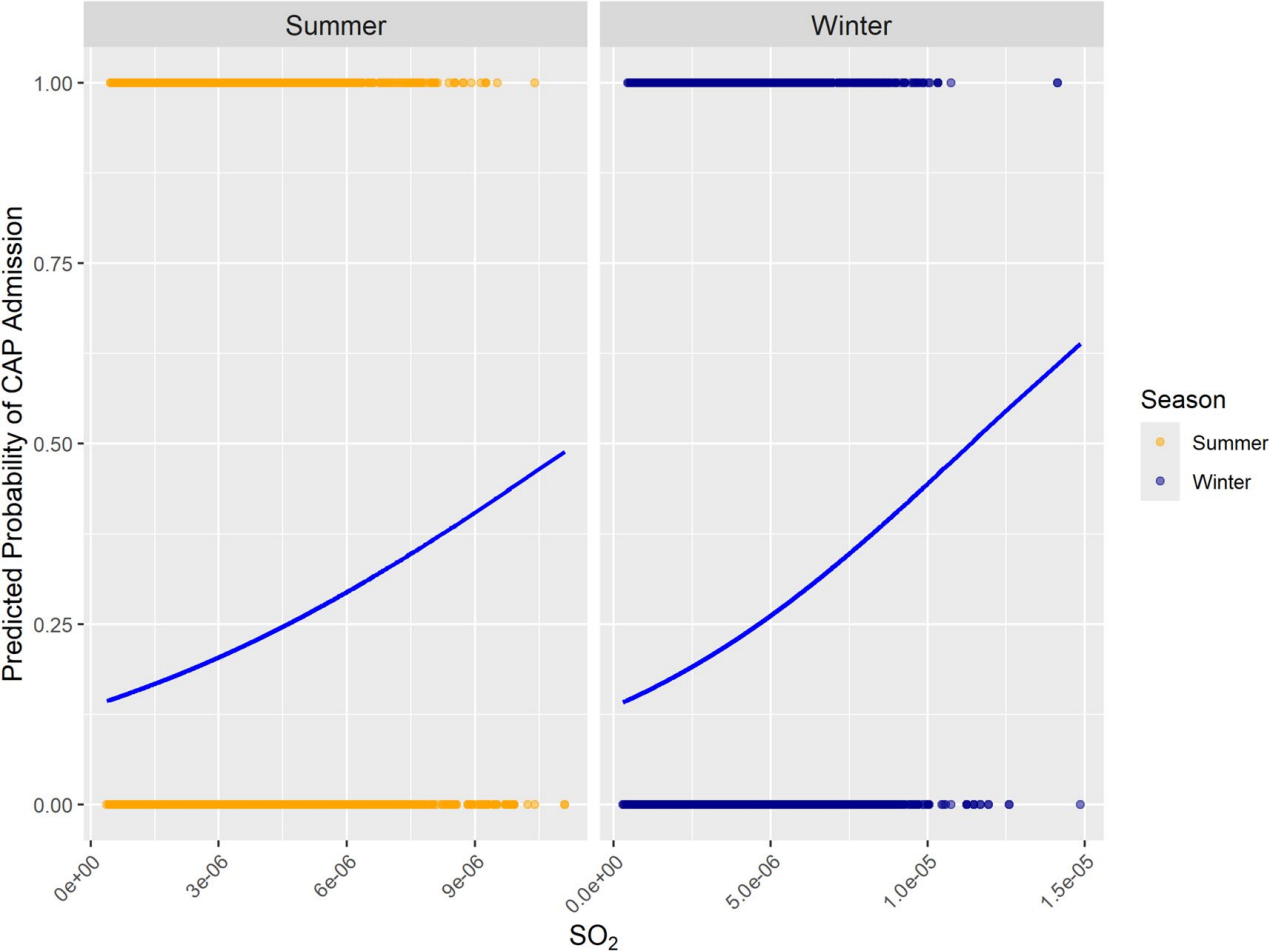
Predicted probabilities of CAP hospitalizations associated with absolute values of SO_2_ during winter and summer

In contrast to meteorological variables, the study found that the absolute values of air quality conditions such as CO, NO_2_, PM_2.5_, SO_2_, and total aerosols were better predictors of CAP hospitalizations than changes in these conditions. In the literature, changing air conditions are not examined in the context of CAP, while numerous studies have documented the harmful effects of air pollutants on respiratory health (Eguiluz-Gracia et al. 2020; Manisalidis et al. 2020; Lee et al. 2021; Li et al. 2021; Yee et al. 2021; Wang et al. 2022; Moamer et al. 2023). Hence, we seem to be the first to consider the relationship between changes in air quality and CAP hospitalization. We find some positive relationships but a much stronger relevance of the level of air quality. Probably, this is due to the fact that exposure to air pollutants increases vulnerability to infections like CAP independent of what happened the days before. The effects seem rather cumulative: the longer people are exposed to air pollutants, the more likely their respiratory health is harmed. Again, an additional look at the logistic regression lines provides further insights (Fig. 5): In the case of the most important air pollutant SO_2_, the values are higher in the winter period compared to the summer period, leading to more CAP hospitalization in winter. This is probably caused by thermal inversion, which occurs much more frequently in winter and contributes to the higher pneumonia probability in winter.

The robustness checks were done to judge the reliability of the results, which were in most cases confirmed. However, one deviation in the robustness checks deserves some discussion. Dust aerosols (absolute value) show a significant relationship with CAP hospitalization only in rural areas. This may be attributed to the elevated exposure to dust aerosols in urban areas, which may cause the concentration of dust aerosols to vary within a range that is harmful anyway. Another explanation could be the difference in population composition. However, this warrants further research into the urban-rural differences and the dependence of weather and air quality sensitivity on personal characteristics.

A main limitation of our study is that we are only able to approximate the exposure of people to meteorological and air conditions, since we do not know their exact location and whether they have been mainly outdoor or indoor. Furthermore, we do not include information on personal characteristics, comorbidities and medication, which can be expected to influence infection and severity of CAP disease.

## Conclusion

We used data on CAP hospitalizations in Germany (CAPNETZ-cohort) to examine whether the absolute values or the changes in meteorological and air quality conditions are more relevant for these hospitalizations. We find that absolute levels are more relevant in the case of air pollutants and relative humidity, while day-to-day changes are more relevant in the case of maximum temperature and precipitation. The findings have two main implications. Firstly, they inform researchers about the way in which they should include meteorological and air quality conditions in their analyses. Secondly, for the society, the results imply that, concerning CAP, people should be especially warned in case of extreme changes in temperatures and precipitation from one to the next day and not in case of extreme values of these. In contrast, high values are relevant in the case of air pollutants. Of course, it is important to conduct similar studies in other countries with different climates to examine whether our findings are generally valid.

## Electronic supplementary material

Below is the link to the electronic supplementary material.

ESM 1 (PDF 189 KB)

## Abbreviations

AIC: Akaike information criterion.
CAP: Community-acquired pneumonia.
CO: Carbon monoxide.
DWD: German Weather Service (Deutscher Wetterdienst).
ECMWF: European Centre for Medium-Range Weather Forecasts.
NO_2_: Nitrogen dioxide.
O_3_: Ozone.
PM: Particulate matter.
SO_2_: Sulfur dioxide.
SRTM: Shuttle Radar Topography Mission.
