# Supplementary material for: Impact of absolute values and changes in meteorological and air quality conditions on community-acquired pneumonia in Germany
Source: Int J Biometeorol. 2024 Dec 24;69(3):695–702. doi: 10.1007/s00484-024-02839-7 (PMC11861118; doi:10.1007/s00484-024-02839-7)
Supplement: Supplementary file 1 — (PDF 189 KB) [file 484_2024_2839_MOESM1_ESM.pdf]

**Impact of absolute values and changes in meteorological and air quality conditions on community-acquired pneumonia in Germany**

**Supplementary Material**

*Table 1 Information on the hospitals and clinics included in the CAPNETZ clinical dataset used in in this study. The table provides details on the duration, number of cases, geographical coordinates, and altitude.*

| Hospitals/ Outpatient Clinics | Considered Period | Cases | Coordinates | Altitude |
|---|---|---|---|---|
| Aachen (Uniklinik RHTW) | 2008-2016 | 142 | 50° N, 6° E | 230 m |
| Bad Arolsen (Krankenhaus) | 2011-2016 | 233 | 51° N, 9° E | 290 m |
| Berlin-Charité (Universitätsmedizin Berlin) | 2005-2017 | 619 | 52° N, 13° E | 35 m |
| Berlin-Heckeshorn (Lungenklinik) | 2003-2017 | 1436 | 52° N, 13° E | 49 m |
| Berlin-Neukölln (Vivantes Klinikum Neukölln) | 2009-2016 | 102 | 52° N, 13° E | 58 m |
| Bochum (Augusta Kliniken) | 2003-2017 | 1086 | 51° N, 7° E | 103 m |
| Cottbus (Carl-Thiem Klinikum) | 2009-2017 | 400 | 51° N, 14° E | 91 m |
| Dresden (Universitätsklinikum Carl Gustav Carus) | 2013-2016 | 56 | 51° N, 13° E | 113 m |
| Hannover (Lungenpraxis Kock) | 2005-2016 | 600 | 52° N, 9° E | 79 m |
| Heidelberg (Thoraxklinik) | 2016-2017 | 11 | 49° N, 8° E | 127 m |
| Jena (Universitätsklinikum Jena) | 2011-2016 | 72 | 50° N, 11° E | 195 m |
| Köln (Universitätsklinikum) | 2003-2004 | 186 | 51° N, 7° E | 56 m |
| Leipzig (Robert Koch-Klinik) | 2006-2009 | 119 | 51° N, 12° E | 128 m |
| Lübeck (Infektiologie Universitätsklinikum) | 2003-2016 | 1260 | 53° N, 10° E | 28 m |
| Lüdenscheid (Lüdenscheid Klinikum) | 2003-2008 | 661 | 51° N, 7° E | 452 m |
| Würzburg Mitte (Missioklinik) | 2003-2004 | 104 | 49° N, 10° E | 234 m |
| Paderborn (Brüderkrankenhaus St. Josef) | 2009-2016 | 315 | 51° N, 8° E | 174 m |
| Regensburg (Klinikum der Universität Regensburg) | 2010-2011 | 6 | 48° N, 12° E | 427 m |
| Rostock (Institut für Klinische Pharmakologie) | 2008-2010 | 61 | 54° N, 12° E | 21 m |
| Rotenburg (Agaplesion Diakonieklinikum) | 2003-2017 | 1568 | 53° N, 9° E | 24 m |
| Ulm (Universitätsklinikum Ulm) | 2004-2015 | 1156 | 48° N, 9° E | 615 m |
| Magdeburg (Universitätsklinikum Magdeburg) | 2003-2005 | 467 | 52° N, 11° E | 56 m |

*Table 2 Description of the meteorological and air quality conditions analyzed in this study.*

| Condition | Unit | Description | Data |
|---|---|---|---|
| **Meteorology** | | | |
| Air pressure | hPa | Average daily air pressure | DWD |
| Relative Humidity | % | Daily average relative humidity | DWD |
| Maximum temperature | °C | Daily maximum temperature | DWD |
| Standardized precipitation exceeding fixed percentiles | dimensionless | Standardized daily precipitation amount over the grid point's 99th percentile of wet days (daily precipitation ≥ 1 mm). Values are decimal, ranging between 0-10 (99th percentile). These values may be used to detect and rank extreme precipitation events. | Copernicus Climate Change Service |
| **Air quality** | | | |
| Maximum total column* CO | kg kg-1 | Daily maximum total column of CO | ECMWF |
| Total column* $NO_2$ | kg kg-1 | Daily maximum total column of $NO_2$ | ECMWF |
| Total column* $O_3$ | $kg/m^2$ | Daily maximum total column of $O_3$ | ECMWF |
| Total column* $SO_2$ | kg kg-1 | Daily maximum total column of $SO_2$ | ECMWF |
| PM 2.5 concentrations | $kg/m^3$ | Daily maximum of particulate matter < 2.5 µm (aerosol) | ECMWF |
| Dust aerosol optical depth** | dimensionless | Daily maximum aerosol optical depth at 550 nm for dust aerosols | ECMWF |
| Total aerosol optical depth** | dimensionless | Daily maximum aerosol optical depth at 550 nm for total aerosols | ECMWF |

* Total column refers to the total amount of the selected variable in a column of air extending from the surface of the Earth to the top of the atmosphere.

**The aerosol optical depth measures the amount of light lost due to the presence of aerosols on a vertical path through the atmosphere.
